# The Interplay Between Autophagy and Senescence in Anthracycline Cardiotoxicity

**DOI:** 10.1007/s11897-021-00519-w

**Published:** 2021-06-03

**Authors:** Michele Russo, Enrico Bono, Alessandra Ghigo

**Affiliations:** Department of Molecular Biotechnology and Health Sciences, Molecular Biotechnology Center, University of Torino, Via Nizza 52, 10126 Torino, Italy

**Keywords:** Cardiotoxicity, Autophagy, Senescence, Doxorubicin, Cardio-Oncology

## Abstract

**Purpose of Review:**

Doxorubicin (DOXO) is a highly effective chemotherapeutic drug employed for the treatment of a wide spectrum of cancers, spanning from solid tumours to haematopoietic malignancies. However, its clinical use is hampered by severe and dose-dependent cardiac side effects that ultimately lead to heart failure (HF).

**Recent Findings:**

Mitochondrial dysfunction and oxidative stress are well-established mechanisms of DOXO-induced cardiotoxicity, although recent evidence suggests that deregulation of other biological processes, like autophagy, could be involved. It is increasingly recognized that autophagy deregulation is intimately interconnected with the initiation of detrimental cellular responses, including autosis and senescence, raising the possibility of using autophagy modulators as well as senolytics and senomorphics for preventing DOXO cardiotoxicity.

**Summary:**

This review aims at providing an overview of the signalling pathways that are common to autophagy and senescence, with a special focus on how the relationship between these two processes is deregulated in response to cardiotoxic treatments. Finally, we will discuss the potential therapeutic utility of drugs modulating autophagy and/or senescence for counteracting DOXO cardiotoxicity.

## Introduction

Anthracyclines are a well-known class of antibiotics commonly used in the clinic as chemotherapy drugs because of their cytotoxic proprieties. Doxorubicin (DOXO) is the most potent member of the family that includes daunorubicin, epirubicin and idarubicin. Although several new-generation targeted therapies have been developed for the treatment of cancer, DOXO still remains the first-line treatment against a broad spectrum of solid tumours and haematopoietic malignancies. However, the clinical use of DOXO is often hampered by the onset of dose-dependent cardiovascular complications, usually occurring within 1 year from the completion of the treatment [1]. In the last decade, with the blooming of cardio-oncology, different strategies aimed at limiting or preventing the cardiotoxic effects of anthracyclines are being developed, including the use of antioxidant molecules designed to block DOXO-related oxidative stress [2•]. Unfortunately, although these compounds have proven successful in experimental animal models, they failed to show significant clinical benefits. The only drug approved by the FDA for the treatment of anthracycline cardiotoxicity is the anti-oxidant and iron chelator dexrazoxane, whose efficacy and safety have long been debated [3].

The failure of clinical trials with antioxidants has indicated that other molecular mechanisms besides oxidative stress likely account for the cardiac side effects of anthracyclines. Among the alternative mechanisms that have been extensively investigated in the last 5 years is autophagy, a cellular recycling process whose alteration has already been linked to a number of cardiovascular diseases (CVDs) [4]. Although the role of autophagy in DOXO cardiotoxicity has long been controversial, mainly as a consequence of the diversity of the experimental models used [5•], the prevailing view is that autophagy is impaired by DOXO and that means of restoring adequate levels of autophagy could be exploited to prevent or treat cardiotoxicity [6••]. Of note, drugs modulating autophagy have already been tested as potential treatments for other pathological conditions, including neurodegenerative disorders, metabolic diseases, tumorigenesis, as well as ageing-related CVDs [7, 8], and could be repurposed for mitigating DOXO-induced cardiotoxicity [9•].

Intriguingly, deregulation of autophagy, along with other hallmarks of anthracycline-mediated injury, like activation of the DNA damage response and epigenetic reprogramming, is one of the key features of senescence. This is increasingly recognized as a stress and damage response with a clear pathogenic role, also occurring in non-dividing and terminally differentiated cells, like cardiomyocytes [10••]. Accordingly, emerging evidence suggests that persistence of senescent cells could be a major cause of cardiotoxicity and that elimination of such cell subpopulations could help limit the cardiac side effects associated to the use of doxorubicin [11••, 12].

Here, we will summarize how autophagy and senescence are interconnected and how their deregulation could contribute to anthracycline cardiotoxicity. Finally, we will speculate on how drugs targeting autophagy, senescence, or both could be exploited for preventing and treating cardiac complications of anthracycline-based anti-cancer regimens.

## Autophagy at the Crossroad of Cell Survival and Senescence in DOXO-Mediated Cardiotoxicity

**Fig. 1:**
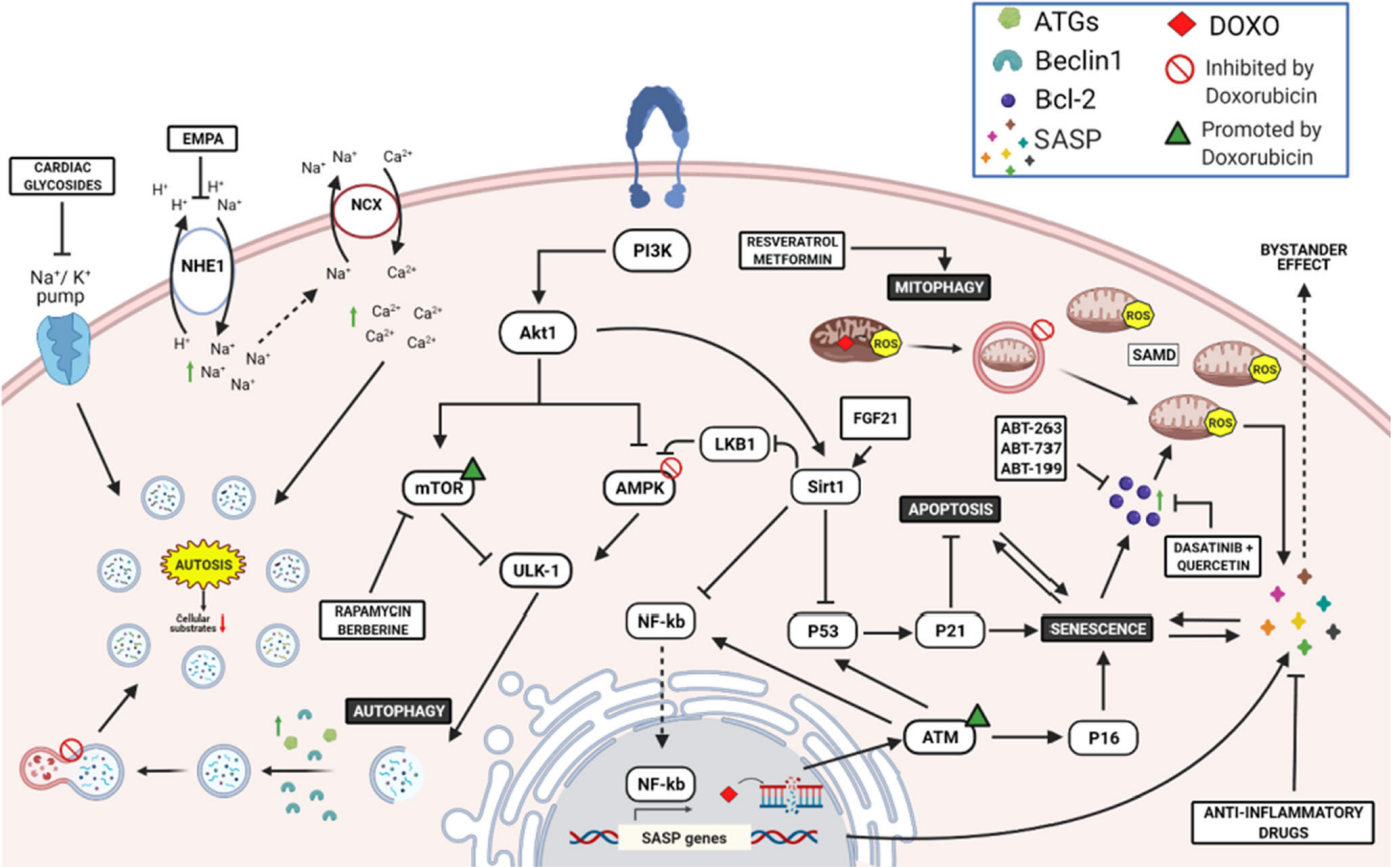
Mechanisms of DOXO-induced senescence. DOXO-mediated DNA damage triggers activation of the p53/21 axis, promoting transition to senescence. Senescence is later endorsed by activation of p16^INK4a^ pathways, favouring pro-inflammatory SAPS factors expression, which, in turn, are exaggerated by an accumulation of dysfunctional mitochondria and ROS production (SAMD). Thus, administration of senomorphics and senolytics provides beneficial effects, restoring mitophagy or specifically killing/depleting senescent cells, respectively. On the other hand, DOXO-mediated blockade of autophagosome-lysosome fusion leads to an aberrant accumulation of autophagosomes, triggering autosis. Finally, EMPA and cardiac glycosides ameliorate intracellular ion homeostasis, restoring autophagic flux. This figure was created with BioRender.com

Autophagy is an essential process for the maintenance of cellular homeostasis. Under physiological conditions, autophagy is maintained at low levels, being increased in response to a wide spectrum of stress stimuli [13]. Autophagy is characterized by the formation of the autophagosome, a spherical structure with double-layered membranes that engulf dysfunctional cellular components, including organelles, allowing their elimination through the fusion with the lysosome. This structure is referred to as the autophagolysosome and represents the functional unit of the autophagy machinery. The entire process consists of five steps (initiation, nucleation, elongation, maturation and degradation) and requires the recruitment of autophagy-related genes (ATG) proteins [14]. The process is controlled by AMPK and mTOR that are the main upstream regulators which promote and inhibit ULK-1 activity, respectively (Fig. 1). ULK1, also known as ATG1, is the protein that triggers autophagy initiation by phosphorylating and positively regulating Beclin-1 [15]. On the other hand, Beclin1 activity is suppressed by Bcl2 after pro-survival stimuli, acting as an early check point of autophagy regulation [16]. These steps are crucial to the assembly of the ULK1/Beclin-1/Atg14L/Vps34/Vps15 complex that is required to the autophagosome formation. The vacuolar protein sorting 34 (Vps34) triggers the production of phosphatidyl inositol 3-phosphate (PI3P) which, in turn, promotes the recruitment of further ATG proteins, such as ATG18, ATG20, ATG21 and ATG24, ultimately inducing phagosome elongation [17]. Autophagosome maturation is mediated by microtubule-associated protein 1 light chain 3 (LC3) that, in cells, is found in a cytosolic (LC3-I) and transmembrane (LC3-II) form. LC3-II is the result of ATG4-mediated proteolytic cleavage at the C-terminus of LC3-I and subsequent conjugation of phosphatidylethanolamine (PE) [18]. These modifications generate an insoluble domain that enables the intercalation of LC3-II into the double membrane of the native phagosome, driving the recruitment in situ of proteins carrying an LC3-II-interacting domain (LIR) [19]. The most studied protein bearing a LIR domain is sequestosome-1, also referred to as p62, that is responsible of selecting ubiquitinated proteins that are destinated to degradation [20]. Other proteins carrying LIR domains, including NBR1, TRAF2 and SMURF1, can participate to the process [21]. This mechanism is highly conserved within eukaryotic cells, and despite the evolutionary divergencies described among species in autophagy-related genes [22], they show high similarity in mammals. For this reason, alterations of the autophagic flux have been found in many pathological conditions [23].

In the heart, the process of autophagy ensures a good plasticity of the tissue in response to a wide range of stressors. In fact, the replacement of damaged organelles, degradation of long-lived/misfolded proteins and mobilization of alternative energetic sources through autophagy are necessary for the homeostasis of terminally differentiated/post-mitotic cells, such as cardiomyocytes, where cellular replication is not an option [24]. On the other hand, chronic activation of autophagy is associated with maladaptive cardiac responses, such as ischemia/reperfusion (I/R) injury and haemodynamic stress [25–27].

The role of autophagy in DOXO-induced cardiomyopathy has been recently investigated and has led to conflicting results [5, 28]. Lu et al. first described that DOXO injection in rats increases Beclin1 expression and autophagosome formation [29]. Additional studies confirmed this observation, showing an increased expression of autophagy-related markers, such as ATG proteins, LC3-II and p62 in DOXO-treated hearts [30–35]. In contrast, other studies support the view that DOXO treatment abolishes autophagy through the upstream inhibition of AMPK, the upregulation of mTOR and the diminished expression of autophagy-related genes [36–41]. These inconsistent conclusions are partly explained by technical variables, primarily the lack of accurate analysis of the autophagic flux in many studies that draw their conclusions simply on the basis of an analysis of the expression levels of proteins involved in autophagosome formation, such as LC3-II and p62. Moreover, the use of different protocols for inducing cardiotoxicity in vivo, including the use of different DOXO doses and different functional endpoints, further complicates the interpretation of the results [5•]. Despite these uncertainties, the prevailing view is that DOXO impairs autophagy and, in turn, the proper disposal of injured mitochondria that ultimately accumulate and constitute a primary source of oxidative stress [6••]. Of note, mitochondria-derived ROS induce extensive damage of both genomic and mitochondrial DNA, ultimately leading to the activation of the p53 pathway and the DNA damage response (Fig. 1) [42••]. Whether p53 activation is beneficial or detrimental is still debated. On the one hand, p53 upregulation might preserve mitochondrial DNA integrity, preventing long-term cardiotoxicity [43, 44]. On the other hand, acute activation of p53 in the heart suppresses the expression of peroxisome proliferator-activated receptor gamma, coactivators 1 alpha and beta (PGC-1α and PGC-1β), two master promoters of mitochondria biogenesis [45]. In agreement with this latter study, p53-deficient mice show preserved cardiac function after DOXO treatment, likely as a consequence of preserved activity of anti-oxidant enzymes [46]. The study of Hoshino et al. suggests that an additional mechanism behind the cardioprotection of p53 knock-out mice could be the preservation of mitochondrial autophagy since DOXO-mediated activation of p53 exaggerates ROS production and in turn impairs Parkin-mediated mitophagy. Of note, p53-deficient hearts are protected not only against DOXO-induced cardiotoxicity but from the functional cardiac decline observed with age, a process that is mechanistically linked to induction of senescence [47]. Therefore, p53-dependent disturbance of mitophagy and clearance of damaged mitochondria is a key process guiding the fate of cardiomyocytes towards survival, apoptosis or senescence [6, 45].

In further support of a functional connection between the processes of autophagy and senescence, it has been shown that autophagy critically controls the turnover and the cellular localization of stress-induced senescence marker proteins, such as p16^INK4a^. Coryell et al. demonstrated that besides being regulated at transcriptional level, p16^INK4a^ activity is finely tuned by the autophagy-lysosome pathway. Activation of this mechanism blocks p16^INK4a^ translocation from the cytoplasm to the nucleus and inhibits the transition to senescence. Of note, this mechanism is impaired in response to several stress stimuli, including cell starvation, oxidative stress as well as chemotherapy treatment, all inhibiting p16^INK4a^ degradation and promoting senescence [48].

The above-mentioned mechanism may explain the accumulation of senescent cells observed in cancer patients undergoing radiotherapy/chemotherapy regimen and associated with high risk of premature age-related cardiovascular complications [49••]. Clinical studies reported that patients receiving chemotherapy treatment have accumulation of p16^INK4a^-positive cells in different tissues, including the heart [50–53], which is detected within 1 year from treatment interruption [52–54].

In the following paragraph, we will describe the role of senescence in cardiac cells and how this process is interconnected with autophagy regulation.

## DOXO Induces Senescence Through Mitophagy Dysregulation and Increased Oxidative Stress

Discovered in 1961, senescence was originally identified as an age-dependent mechanism associated with reduction of telomerase activity in replicant cells [55]. Senescent cells, which can be identified based on the increased expression of senescence-associated-β-galactosidase (SA-β-gal), are characterized by high resistance to apoptotic and growth stimuli, associated to high expression of the pro-survival protein Bcl-2 and of the cyclin-dependent inhibitor of the cell cycle p16^INK4A^, respectively [56, 57]. Interestingly, activation of the p53/p21 axis is described as an early marker of senescent transition, while p16^INK4A^ is required later to maintain the senescent phenotype [58] and acts as a point of no return (Fig. 1) [59]. Beyond cell cycle arrest, other features of senescent cells include changes in the morphology of the main cellular compartments, increased mitochondria contents and metabolic rearrangements [60].

Interestingly, the definition of senescence has been recently reconsidered, leading to the emerging concept that senescence is a cellular stress response triggered by different stressors, spanning from DNA damage, mitochondrial dysfunction and oxidative stress, all key hallmarks of chemotherapy- and radiotherapy-mediated cell injury [10••, 61]. Therapy-induced senescence (TIS) indeed represents one of the mechanisms whereby anticancer agents like anthracyclines and ionizing radiation kill tumour cells [49••, 62, 63]. However, recent findings highlight that TIS may be detrimental, promoting aggressive tumour relapse and negatively affecting normal cells [11••]. In agreement with this notion, accumulation of senescent cells in the heart has been proposed as a major cause of the late-onset cardiovascular complications observed in patients undergoing radiotherapy and anthracycline chemotherapy [49••].

Although senescence was originally described as restricted to replicant cells, this cellular response has been observed also in post-mitotic cells, such as neurons and cardiomyocytes. The pathophysiological relevance of this postmitotic cell senescence (PoMiCS) is still incompletely understood. On the one hand, acute stress-induced premature senescence (SIPS) might be beneficial in tissue healing and repair [64, 65], particularly in organs with limited regenerative capacity, such as the brain and the heart [10••, 61]. On the contrary, senescent cells may have a pathogenic role that is linked to their ability to secrete a number of pro-inflammatory factors, including cytokines, chemokines, growth factors and metalloproteinases, overall referred to as senescence-associated secretory phenotype (SASP) [66]. On the one side, these secreted factors promote short-term tissue homeostasis and repair, while on the other side, they may cause maladaptive tissue remodelling and tumorigenesis due to tissue inflammation and immune cell suppression [67•]. Additionally, SASP factors exert a paracrine action, referred to as ‘bystander effect’, inducing senescence of neighbour cells both in vitro [68] and in vivo [69, 70•] (Fig. 1).

In DOXO cardiotoxicity, this secretory phenotype is likely activated by ROS generation that ensues from the abnormal accumulation of dysfunctional mitochondria after anthracyclines exposure (Fig. 1) [67•]. Consistently with this view, Correia-Melo et al. demonstrated that mitophagy-dependent depletion of mitochondria in senescent cells has reduced production of SASP without affecting cell cycle machinery [71, 72]. This supports the hypothesis that mitochondrial ROS are key modulators of SAMD phenotype, promoting NF-κB-dependent transcription of SASP factors (Fig. 1) [73].

In further support of the notion that autophagy inhibition is a trigger of senescence, autophagy re-activation, through either dietary restriction or small molecules, has been demonstrated to improve mitochondrial function [74], counteract the SAMD phenotype [71, 75] and deplete senescent cells in different tissues [76] (Table 1). On these grounds, one may speculate that autophagy activators could be exploited for preventing the induction of senescence in DOXO-treated hearts. In the following paragraph, we will discuss how drugs targeting the process of autophagy can indirectly contribute to block the process of senescence in response to DOXO.

**Table 1:** Proposed mechanisms of senotherapeutic molecules in DOXO-induced premature senescence

Class of molecule	Type of treatment	Biological effects	References
Senomorphs	Resveratrol	Preserves mitochondrial activity and prevents cAMP degradation through activation of SIRT1/PI3K/Akt axis.	[77]
	Metformin	Promotes AMPK activation via phosphorylation of Ser633 and Ser1177.	[78]
	Rapamycin	Positive autophagy modulation by mTOR inhibition and AMPK activation.	[9•]
	Berberine	Inhibits mTOR signalling and increases DOXO detoxification.	[79]
Senolytics	Quercetin	Positive metabolic modulation, iron chelating activity, apoptosis inhibition, prevention of tissue remodelling and reduction of oxidative stress and inflammation. Increases cardioprotective effect of Losartan. Inhibits Bcl-2 in tumour and senescent cells.	[49, 80–83]
	Dasatinib + Quercetin	Increased killing of p16^+^ and p21^+^ senescent cells.	[84]
	ABT263, ABT737	Eliminates p16^+^ positive cells, inhibiting the activity of Bcl-2 and Bcl-xL.	[11••]
	ABT-199	Prevents ABT-263-mediated thrombocytopenia manifestation in patients through specific inhibition of Bcl-2.	[85]
	Cardiac glycosides	Disrupt intracellular concentrations of Na^+^, K^+^ and H^+^ in senescent cells.	[86]

## Senomorphic Effect of Autophagy-Inducing Molecules

Senomorphics are a class of molecules that revert the morphology of senescent cells. These agents specifically target pathways required for the maintenance of the senescent state, without interfering with the cell-cycle machinery. Of note, the majority of these molecules are autophagy modulators and thus inhibit senescence indirectly through reactivation of autophagy. These include mTOR inhibitors and AMPK activators, the master regulators of autophagy. Consistently with this, cardiac inhibition of mTOR showed beneficial effect on SIPS, inducing parkin-mediated mitophagy and enhancing mitochondria turn-over [87•]. Moreover, mTOR inhibitors, such as Rapamycin and Berberine, positively modulate AMPK-induced autophagy (Fig. 1) [9•]. Particularly, experimental results in rodents showed that Berberine pre-treatment prevent DOXO-induced cardiac damage [79, 88, 89]. However, the beneficial effect of Berberine is due to inhibition of doxorubicin metabolism and reduced accumulation of its toxic metabolite doxorubicinol in the heart [79], rather than AMPK activation [89]. Of note, the effect of AMPK modulators, such as metformin, is reported as an attractive strategy to prevent the cardiometabolic impairments provoked by DOXO [90•]. Turdi and colleagues showed that low-dose Metformin exerts an AMPK-mediated beneficial effect on ageing hearts, improving mitochondrial function and diminishing ROS production (Fig. 1) [78]. Indeed, AMPK plays a key role in response to reduced ATP levels caused by dysfunctional mitochondria in senescent cells, suppressing the NF-κB-mediated SASP production and improving cell survival [91]. Similarly, experimental evidence showed the cardioprotective effects of Fibroblast growth factor 21 (FGF21) against DOXO-induced cardiomyopathy via activation of SIRT1/LKB1/AMPK axis. One mechanistic study revealed that FGF21 treatment enhances SIRT1-mediated inhibition of LKB1, an upstream negative modulator of AMPK, improving cardiac inflammation and mitochondrial respiration (Fig. 1) [92]. SIRT1 is a NAD^+^-dependent histone deacetylase that regulates p53 activity and inhibits the transcriptional activity of NF-kB, whose function is compromised in cells with reduced NAD^+^/NADH ratio (Fig. 1) [93, 94]. The reduction in NAD^+^ metabolism represents an important feature of cells undergone SAMD and drives SASP release [95]. To this aim, NAD^+^ supplementation and SIRT1 agonists were proposed as an optimal strategy in counteracting the pro-inflammatory senescence-associated secretome [95, 96]. Experimental evidence in vivo showed that resveratrol ameliorates cardiac function through activation of SIRT1/PI3K/Akt axis, preserving mitochondrial activity and preventing cAMP degradation [77]. Although the role of SIRT1 was not further investigated by Matsumura et al., this should delineate the beneficial effect of Resveratrol in response to the DOXO-mediated upregulation of p53 [97, 98]. Indeed, SIRT1 has been found to induce autophagy independently from the inhibitory effect of p53 and mTOR pathways [99, 100]. Furthermore, oral administration of the natural polyamine Spermidine exerts protective effects, ameliorating cardiac contraction, lowering cardiac hypertrophy and suppressing tissue inflammation. This cardioprotection is lost in mice where autophagy is pharmacologically or genetically abolished [101, 102]. Furthermore, genetic studies should clarify the additive epigenetic impact of Spermidine and Resveratrol, causing their inhibition of histone acetylases and activation of histone deacetylase respectively [101]. Our previous study showed that dietary supplementation of phenylalanine-butyramide, a potent histone deacetylase inhibitor, protects from DOXO-induced mitochondrial dysfunction and counteracts oxidative stress [103]. Overall, these findings suggest that improvement of the autophagic machinery can positively affects heart functions reverting the DOXO-associated SAMD phenotype.

## The New Frontier of Senolytics in DOXO Cardiotoxicity

Senolytics are a new generation of compounds devised to specifically eliminate senescent cells, acting on pathways that protect senescent cells from apoptosis (Table 1). Compared to senomorphics, senolytics exert their pharmacological effect at lower concentrations, thus being well tolerated in animal models and holding great promise for clinical translation. Senolytic agents include natural compounds, such as quercentin, fisetin, piperlongumine, curcumin and cardiac glycosides as well as anticancer molecules, like dasatinib [104••]. Only three of them have so far been tested in preclinical models for their ability to deplete senescent cells in response to chemotherapy treatment and include dasatinib, quercetin and ABT263 (Navitoclax) [49••].

ABT263, along with A1331852 and A1155463, is an inhibitor of the anti-apoptotic proteins BCL-2 and BCL-xL. Demaria and co-workers demonstrated that ABT263 administration efficiently eliminates senescent cells after DOXO injection in transgenic p16-3MR tumour-bearing mice, in which p16^INK4^ positive cells can be detected using live imaging techniques. ABT263-mediated depletion of senescent cells results in an improvement of the health status of animals undergoing chemotherapy regimen, associated with a reduction in SASP-induced systemic inflammation, diminished organ toxicity and preserved cardiac function [11••]. However, although ABT263 was designed to ameliorate the efficacy of the analogue molecule ABT-737, its inhibition of Bcl-xL causes thrombocytopenia. The Bcl-2 selective inhibitor ABT-199 (Venetoclax) has been developed to overcome this limitation, and results from phase I clinical trial showed encouraging results (Fig. 1) [85].

Another senolytic agent is quercetin, a plant-derived flavonoid that has been tested for its antineoplastic activity alone or in combination with DOXO. Preliminary experiments in vitro demonstrated that quercetin increases the antineoplastic activity of DOXO at low dose and protects non-tumoral cells [105, 106]. Several studies are ongoing to investigate the molecular mechanisms underlying the protective effects of quercetin against DOXO toxicity, which could include restoration of cellular metabolism, iron chelating activity, apoptosis inhibition, prevention of tissue remodelling and reduction of oxidative stress and inflammation [80, 81]. Additionally, quercetin administration augments the effect of cardioprotective molecules, such as losartan and resveratrol, in models of DOXO-induced cardiotoxicity [82, 83]. Surprisingly, inhibition of the anti-apoptotic factor Bcl-2 is the main effect triggered by quercetin in cancer and senescent cells (Fig. 1) [49••]. Therefore, further studies are needed to elucidate the cell tropism of quercetin. Recently, a clinical trial showed that combined administration of quercetin and dasatinib reduces the number of senescent cells in patients with diabetic kidney disease [84]. Since the first article about senolytic agents was published in 2015 [107], this finding is the first evidence of their efficacy in humans and opens a new promising scenario on their introduction into clinical practice [108••].

Interestingly, cardiac glycosides, such as ouabain and digoxin, exert a potent senolytic activity by virtue of their ability to antagonize the Na^+^/K^+^-ATPase (Table 1) (Fig. 1). Indeed, senescent cells are more susceptible than normal cells to intracellular alterations of Na^+^, K^+^ and H^+^ concentrations [86]. Additionally, cardiac glycosides synergize with chemotherapy and increase the killing of tumoral senescent cells through the expression of the pro-apoptotic protein of the Bcl2-family, NOXA [109].

In addition to selective killing of senescent cells, ouabain also inhibits autosis, a recently described form of autophagy-related cell death that is mediated by the Na^+^/K^+^-ATPase pump (Fig. 1) [110, 111]. The role of autosis in the heart is still poorly characterized and limited to few indications coming from studies conducted in animal models of ischemia/reperfusion (I/R) injury. Nah and colleagues showed that administration of ouabain protects from autosis-induced I/R damage in humanized Na^+^/K^+^-ATPase-knock-in mice. However, although autosis-mediated cell death was initially associated to an increased autophagic flux, treatment with a selective autophagy inhibitor, bafilomycin A1, did not prevent the autotic process. This led to the hypothesis that cardiomyocytes that undergo autosis-dependent cell death may have their cellular functions compromised as a consequence of an excessive autophagosome formation (Fig.1). Indeed, the high amount of material required to assemble the double-membrane structure of autophagosomes may deprive cells of substrates necessary for building fundamental cellular organelles, such as mitochondria, endoplasmic reticulum, Golgi apparatus and plasma membrane [112]. Similarly, modulation of ion concentrations by empagliflozin, an inhibitor of the Na^+^/H^+^ exchanger 1 (NHE1), prevents intracellular Na^+^ and Ca^2+^ overload and restores the autophagic flux, ultimately counteracting the deleterious activation of autosis in an animal model of I/R (Fig.1) [113]. Whether autosis is involved in chemotherapy-induced cardiotoxicity represents a new relevant field of investigation [114].

## Conclusion and Future Perspectives

Overall, studies summarized herein demonstrate that autophagy deregulation represents a major mechanism underlying the cardiac adverse effects of chemotherapy, primarily anthracyclines. Intriguingly, recent findings have revealed how aberrant autophagy can trigger different maladaptive cellular responses, including senescence [115] and autosis [111]. On these bases, strategies aimed at restoring a physiological and balanced autophagic flux could indirectly act by preventing the initiation of those detrimental events and could be exploited for counteracting the cardiac side effects of anthracycline-based therapies. Furthermore, drugs directly favouring the elimination of senescent cells (senolytics) or reverting the senescent phenotype of cardiac cells (senomorphics) could be similarly exploited for the treatment of cardiotoxicity. Nevertheless, additional studies in preclinical models are required to conclusively demonstrate the feasibility of this latter approach.
